# Supplementary material for: FastqPuri: high-performance preprocessing of RNA-seq data
Source: BMC Bioinformatics. 2019 May 3;20:226. doi: 10.1186/s12859-019-2799-0 (PMC6500068; doi:10.1186/s12859-019-2799-0)
Supplement: Supplementary file 2 — Archive of FastqPuri. Archive containing all files needed to install and run FastqPuri v1.0.6. Date stamp March 22, 2019. (GZ 47,819 kb) [file 12859_2019_2799_MOESM2_ESM.gz › FastqPuri-1.0.6/html/struct__iparam__makeBloom.html]

FastqPuri: \_iparam\_makeBloom Struct Reference


FastqPuri

Public Attributes |
List of all members

\_iparam\_makeBloom Struct Reference

contains makeBloom input parameters
More...

`#include <init_makeBloom.h>`

|  |  |
| --- | --- |
| Public Attributes | |
| char \* | inputfasta |
|  | |
| char | filterfile [MAX\_FILENAME] |
|  | |
| char | paramfile [MAX\_FILENAME] |
|  | |
| int | kmersize |
|  | |
| int | hashNum |
|  | |
| double | falsePosRate |
|  | |
| uint64\_t | bfsizeBits |
|  | |
| uint64\_t | nelem |
|  | |

## Detailed Description

contains makeBloom input parameters

Note
:   nelemen will be computed once the fasta file is read and loaded.

## Member Data Documentation

## ◆ bfsizeBits

uint64\_t \_iparam\_makeBloom::bfsizeBits

bloom filter size (bits)

## ◆ falsePosRate

double \_iparam\_makeBloom::falsePosRate

false positive rate

## ◆ filterfile

char \_iparam\_makeBloom::filterfile[MAX\_FILENAME]

filter file path

## ◆ hashNum

int \_iparam\_makeBloom::hashNum

number of hash functions used to construct the filter

## ◆ inputfasta

char\* \_iparam\_makeBloom::inputfasta

fasta input file

## ◆ kmersize

int \_iparam\_makeBloom::kmersize

kmer size (number of elements)

## ◆ nelem

uint64\_t \_iparam\_makeBloom::nelem

number of elements that the bloomfilter will contain

## ◆ paramfile

char \_iparam\_makeBloom::paramfile[MAX\_FILENAME]

param file path

---

The documentation for this struct was generated from the following file:

- include/init\_makeBloom.h


---

Generated on Mon Mar 19 2018 23:42:01 for FastqPuri by  

 1.8.14
